# Effects of intensive care unit quality assessment on changes in medical staff in medical institutions and in-hospital mortality

**DOI:** 10.1186/s12960-024-00893-1

**Published:** 2024-02-02

**Authors:** Seungju Kim, Gui Ok Kim, Syalrom Lee, Yong Uk Kwon

**Affiliations:** 1 Department of Health System, College of Nursing, The Catholic University of Korea, 222, Banpo-Daero, Seocho-Gu, Seoul, 06591 Republic of Korea; 2 Research Institute for Hospice/Palliative Care, The Catholic University of Korea, Seoul, Republic of Korea; 3 Department of Quality Assessment, Health Insurance Review and Assessment Service, Wonju, Republic of Korea; 4 Healthcare Review and Assessment CommitteeHealth Insurance Review and Assessment Service, Wonju, Republic of Korea

**Keywords:** Quality assessment, Quality of care, In-hospital mortality, Intensive care unit

## Abstract

**Background:**

Quality assessments are being introduced in many countries to improve the quality of care and maintain acceptable quality levels. In South Korea, various quality assessments are being conducted to improve the quality of care, but there is insufficient evidence on intensive care units (ICUs). This study aims to evaluate the impact of ICU quality assessments on the structural indicators in medical institutions and the resulting in-hospital mortality of patients.

**Methods:**

This study used data collected in the 2nd and 3rd ICU quality assessments in 2017 and 2019. A total of 72,879 patients admitted to ICUs were included during this period, with 265 institutions that received both assessments. As for structural indicators, changes in medical personnel and equipment were assessed, and in-hospital deaths were evaluated as patient outcomes. To evaluate the association between medical staff and in-hospital mortality, a generalized estimating equation model was performed considering both hospital and patient variables.

**Results:**

Compared to the second quality evaluation, the number of intensivist physicians and experienced nurses increased in the third quality evaluation; however, there was still a gap in the workforce depending on the type of medical institution. Among all ICU patients admitted during the evaluation period, 12.0% of patients died in the hospital. In-hospital mortality decreased at the 3rd assessment, and hospitals employing intensivist physicians were associated with reduced in-hospital deaths. In addition, an increase in the number of experienced nurses was associated with a decrease in in-hospital mortality, while an increase in the nurse-to-bed ratio increased mortality.

**Conclusions:**

ICU quality assessments improved overall structural indicators, but the gap between medical institutions has not improved and interventions are required to bridge this gap. In addition, it is important to maintain skilled medical personnel to bring about better results for patients, and various efforts should be considered. This requires continuous monitoring and further research on long-term effects.

## Background 

Owing to the aging population and the increase in chronic diseases, the number of patients with multiple chronic diseases is increasing, which is leading to an increase in intensive care unit (ICU) admissions requiring more specialized treatment [1–4]. While ICU admission is expected to result in better outcomes in the ICU, it can lead to other health problems owing to patient vulnerabilities, including effects from the availability of medical resources, such as facilities and medical staff [5, 6]. Infection is a common health problem that can lead to an increase in in-hospital mortality, and patients admitted to ICUs have a high risk of death even after discharge [7–9]. Therefore, proper quality control in ICUs is important, and better patient outcomes should be achieved through appropriate management.

To address these issues, South Korea is introducing a quality evaluation program in medical institutions. Quality assessment is a value-based incentive system implemented by the Health Insurance Review and Assessment Service (HIRA), in accordance with the National Health Insurance (NHI) Act amended in 2000; incentives or penalties are given to medical institutions according to the results of quality evaluation [10]. This quality assessment is based on Donabedian’s healthcare quality evaluation model and is evaluated in terms of structure, process, and outcomes [11]. The first ICU evaluation was conducted in 2014 for medical institutions above general hospitals, with seven evaluation indicators and six monitoring indicators, and the results were announced in 2016 [12]. According to the HIRA report, the nurse-to-bed ratio was 1:1.1, equipment and facilities for critically ill patients were 3.6 out of 6 points, and 82.9% of institutions had standardized protocols [12]. In particular, the gap in medical personnel by hospital type was large, with only 32.8% of institutions having intensivist physicians, and the nurse-to-bed ratio was 0.61:1 for tertiary hospitals and 1.19:1 for general hospitals. In addition, more than 50% of medical institutions received a grade of 4 or below, showing a large variance in ICUs among medical institutions.

In previous studies, the introduction of quality assessments had a positive effect on medical institutions [13–17]. Introducing quality assessments in stroke care was associated with improvements in overall structural and process indicators and reductions in patient mortality [13]. Introducing colorectal cancer quality assessments reduced the differences in various indicators among medical institutions [14]. Introducing a quality assessment program in patients with chronic obstructive pulmonary disease was associated with improvements in patient care protocols and reduced readmissions and deaths [15, 17]. However, in one study, even after the introduction of a quality assessment, a gap exists between the ideal goal and the clinical environment in asthma management, indicating the need for improved quality management [18].

Donabedian stated that good structure can lead to better outcomes by increasing the likelihood of receiving good processes [11], meaning that structural changes through ICU quality assessment can lead to better patient outcomes. However, there is insufficient evidence on whether structural changes through quality assessment in the ICU lead to better patient outcomes. This study evaluates the impact of introducing ICU quality assessments on hospital structural aspects and patient outcomes. First, to evaluate the overall impact of quality assessments, changes in structural indicators in medical institutions following the introduction of quality assessment programs were evaluated. Second, the impact of quality assessments and structural indicators on patient outcomes and in-hospital mortality was evaluated, while focusing on the impact of medical personnel on patient outcomes.

## Materials and methods

### ICU quality assessment

The ICU quality assessment was conducted every 2 years from 2017 after the first evaluation in 2014 by the HIRA. The results evaluated in 2017 and 2019 were released in 2018 and 2020, respectively. ICU indicators were developed through three major steps. First, the Korean Society of Critical Care Medicine developed quality indicators, and then the appropriateness of the indicators was then evaluated by experts. Finally, the indicators were finalized through the evaluation committee within HIRA [12]. Fourteen evaluation indicators were included in the two evaluations, including 7 evaluation indicators and 7 monitoring indicators. Detailed evaluation indicators are disclosed by each institution through the HIRA website. In this case, monitoring indicators refer to indicators that are notified to individual institutions but are not disclosed to the public. Seven indicators, which are evaluation indicators, are used to calculate the overall score, and if the number of submitted data is less than 10, even if the medical institution undergoes quality evaluation, it is not included in the overall score calculation. Detailed indicators are shown in Table 1.

**Table 1:** ICU quality assessment indicators

		Indicator	Result
Evaluation indicators (7)	Structure	(1) Number of ICU beds per intensivist physician (2) Number of ICU beds per nurse	Low is good
		(3) Number of ICU specialized equipment and facilities - Arterial blood gas analyzer, portable ventilator, continuous renal replacement therapy, bronchoscopy, independent space for specialists in ICUs, isolation ward - Evaluation standard: if a tertiary general hospital has 6 facilities and equipment and a general hospital has 5, this index is perfect (4) Number of ICU care protocol*: 9 - Hospitalization, discharge, ventilator withdrawal, sedation/delirium, prevention of deep vein thrombosis, prevention of bedsores, mechanical ventilation, prevention of ventilator-related pneumonia, sterilization precautions for central catheter insertion	High is good
	Process	(1) Proportion of patients receiving prophylactic treatment for deep vein thrombosis* (2) Standardized mortality ratio assessment	High is good
	Outcome	Readmission to ICU within 48 h*	Low is good
Monitoring indicators (7)	Structure	(1) Percentage of multidisciplinary care team rounds* (2) Percentage of patients using a ventilator*	
	Process	Perform infection-related bundles	High is good
	Outcome	(1) ICU mortality* (2) Central catheter blood infection rate (3) Incidence of pneumonia in ventilator patients (4) Incidence of urinary tract infections related to urinary catheters	Low is good

^*^Indicates that the index is calculated up to 100%, because it is calculated as a percentage

### Database and data collection

This study used the quality assessment result data from 2017 and 2019, collected by the HIRA. In each assessment, the evaluation period was 3 months, and all adult patients aged 18 years or older who were admitted to the ICU of general hospitals and tertiary hospitals between May and July of the evaluation year were included. Exclusion criteria included hospitals that were closed during the evaluation period, hospitals with fewer than 10 cases, patients with less than 48 h of stay in an ICU, neonatal ICU, pediatric ICU, and burn patients. During the evaluation period, each hospital submitted patient and hospital data according to the indicators required by HIRA, and the quality of each hospital was evaluated through quality evaluation based on this data. The data comprised hospital and patient data; hospital data included the basic characteristics of hospitals, such as hospital type and region. Patient data included variables for measuring quality evaluation indicators, such as the ICU admission date, discharge date, discharge results, and ventilator application. Hospitals for which medical personnel information was not submitted were excluded, and only hospitals that received both the 2nd and 3rd evaluations were included to evaluate the change according to the quality evaluation period. In addition, patients admitted to the ICU at the end of the evaluation were excluded according to the quality evaluation criteria, and patients with missing variables were excluded. Finally, 265 tertiary and general hospitals that received both quality assessments and 72,879 patients who were hospitalized during each assessment period were included.

### Variables

The quality evaluation was divided into the 2nd (2017) and 3rd (2019) evaluation, according to the evaluation period. Quality indicators comprise structure, process, and outcome indicators, and they are classified into evaluation and monitoring indicators depending on whether they are included in the comprehensive score calculation. The overall score includes seven evaluation indicators, and the results are disclosed to the public. Detailed evaluation indicators are presented in Table 1. This study first measured changes in structural indicators such as medical staff, equipment, and protocols to evaluate the effect of introducing quality assessment. Changes in medical personnel included the number of intensivist physicians (full-time and half-day), nurse-to-bed ratio, nursing grade, the number of experienced nurses, and average nursing experience. The nurse-to-bed ratio is measured quarterly and is measured by dividing the number of ICU beds over a 3-month period by the number of nurses working in the ICU during that period. A lower ratio means more nurses in a medical institution, and a higher ratio means fewer nurses. Experienced nurses were defined as those with over 3 years of work experience at the hospital. Furthermore, ICU specialized equipment and facilities, number of ICU care protocols, and number of infection-related bundles were included. There are four infection-related bundles (central catheter, ventilator, urinary catheter, and sepsis), and sepsis-related bundles were excluded in the 3rd quality evaluation; thus, having three bundles corresponds to a perfect score.

The second outcome variable was patient outcomes, which measured in-hospital mortality. For those patients who were repeatedly admitted to an ICU without readmission within 48 h, admissions were measured repeatedly on a case-by-case basis. Ultimately, after checking the discharge results for each case in which the patient was admitted to the ICU, the discharge result was coded as death, and if the death date matched, it was defined as in-hospital death. Patient characteristics included sex (female, male), age, use of ventilator (yes, no), use of central venous line (yes, no), health insurance (NHI, Medicaid, Veteran), and major diagnosis based on ICD-10 code. Hospital characteristics included quality assessment (2nd, 3rd), intensive physician (yes, no), nurse-to-bed ratio, number of experienced nurses, number of ICU specialized equipment, number of ICU care protocols, number of infection prevention bundles, type of hospital (general hospital, tertiary hospital), region (capital city area, metropolitan area, other), and number of beds (< 300 beds, < 500 beds, < 700 beds, < 900 beds, ≥ 900 beds).

### Statistical analysis

The distribution of each categorical variable was examined by analysis of frequencies and percentages, and χ^2^ tests were performed to examine associations with quality assessment or death. *T* test or analysis of variance was performed to compare average values and standard deviations for continuous variables. A generalized estimating equation model was used to assess the impact of quality assessment on patient mortality by simultaneously considering hospital and patient variables. In addition, subgroup analysis according to hospital type was performed to evaluate the relationship between structural indicators and mortality in ICUs. All statistical analyses were performed using the SAS statistical software (version 9.4; SAS Institute, Cary, NC, USA). The statistical significance of the calculated indices was set at *p* < 0.05.

## Results

Table 2 shows the general characteristics of hospitals that received both the 2nd and 3rd quality evaluations. A total of 265 hospitals were included in the 2nd and 3rd quality evaluation, including 43 tertiary hospitals (3rd assessment: 42) and 222 general hospitals (3rd assessment: 223). Approximately 40% of hospitals were operating with less than 300 beds, and most hospitals were located in the capital city area or other areas. In general, the number of medical personnel increased in the 3rd evaluation compared to the 2nd.

**Table 2:** General hospital characteristics according to quality assessment period (Unit: n/M, %/SD)

	Quality assessment				*p*
	2nd		3rd		
*Type of hospital*					
Tertiary hospital	43	(16.2)	42	(15.8)	1.00
General hospital	222	(83.8)	223	(84.2)	
*Intensivist physician*					
Yes	112	(42.3)	131	(49.4)	.117
No	153	(57.7)	134	(50.6)	
*Number of beds*					
< 300	112	(42.3)	108	(40.8)	.990
< 500	61	(23.0)	65	(24.5)	
< 700	33	(12.5)	34	(12.8)	
< 900	33	(12.5)	31	(11.7)	
≥ 900	26	(9.8)	27	(10.2)	
*Region*					
Capital area	100	(37.7)	100	(37.7)	1.00
Metropolitan	63	(23.8)	63	(23.8)	
Other	102	(38.5)	102	(38.5)	
*Number of intensivist physician staffing*					
Full-time	0.92	± 1.82	1.02	± 1.79	.026
Half-time	0.26	± 0.69	0.40	± 0.90	.008
*Number of nurses*					
Nurse-to-ICU bed ratio	0.99	± 0.64	0.96	± 0.76	< .001
Nursing grade	4.28	± 2.34	3.89	± 2.41	< .001
Experienced nurse	22.09	± 29.58	23.94	± 32.64	< .001
Average work experience (year)	4.84	± 2.64	4.87	± 2.49	.253
Total	256		256		

Table 3 shows the differences in structural indicators according to hospital types among the items of the 2nd and 3rd quality evaluations. In the 2nd quality evaluation, there was a difference in the number of full-time intensivist physicians in tertiary general and general hospitals (mean difference: 3.53, *p* < 0.001). In the 3rd quality assessment, the number of full-time intensivist physicians increased, but the gap between general and tertiary hospitals did not decrease (mean difference: 3.55, *p* < 0.001). The nurse-to-ICU bed ratio was 0.5 beds per nurse in tertiary hospitals, but 1 bed per nurse in general hospitals, and this difference was the same in the 2nd and 3rd quality assessment. Regarding the nursing grade based on the nurse-to-ICU bed ratio, tertiary hospitals had an average of one grade, but general hospitals had four grades, showing large differences by hospital type (*p* < 0.001). In particular, the number of nurses with more than 3 years of experience was about seven times higher in tertiary hospitals than in general hospitals. In the second quality assessment, ICU specialized equipment and facilities were evaluated as six for tertiary hospitals and five for general hospitals, and the average number of equipment was 5.5 for tertiary hospitals and 3.2 for general hospitals.

**Table 3:** Results of quality indicators by type of hospital according to the 2nd and 3rd quality assessments (Unit: N/M, %, SD)

	2nd quality assessment					3rd quality assessment				
	Tertiary hospital		General hospital		*P*	Tertiary hospital		General hospital		*p*
*Medical staff*										
Number of intensivist physician staffing										
Full-time	3.88	± 2.70	0.35	± 0.73	< .001	4.00	± 2.45	0.45	± 0.84	< .001
Half-time	0.70	± 1.15	0.18	± 0.52	.005	1.12	± 1.60	0.26	± 0.62	.001
Number of nurses										
Nurse-to-ICU bed ratio	0.55	± 0.08	1.07	± 0.67	< .001	0.50	± 0.07	1.05	± 0.79	< .001
Nursing grade	1.77	± 0.65	4.77	± 2.24	< .001	1.38	± 0.54	4.36	± 2.34	< .001
Experienced nurse	72.05	± 39.52	12.41	± 13.00	< .001	79.21	± 44.35	13.52	± 14.72	< .001
Average work experience (year)	5.79	± 1.52	4.66	± 2.77	< .001	5.51	± 1.24	4.75	± 2.65	.004
*Structure and process indicator*										
ICU specialized equipment and facilities	5.53	± 0.50	3.31	± 1.78	< .001	5.48	± 0.51	3.56	± 1.71	< .001
Number of ICU care protocol (total: 9)	9.00	± 0.00	8.59	± 1.61	< .001	9.00	± 0.00	8.83	± 1.05	.019
Infection-related bundles	3.79	± 0.41	2.85	± 1.47	< .001	3.00	± 0.00	2.71	± 0.85	< .001

Table 4 shows the baseline characteristics of patients admitted during the quality assessment period. A total of 72,879 patients were admitted to the ICU, of which 8671 patients (11.9%) died there. Depending on the quality assessment period, 12.0% of patients died in the second evaluation and 11.8% died in the third evaluation; however, the difference was not significant (*p* = 0.438). Most patients were hospitalized in an ICU with an intensivist physician, and the mortality rate was higher in hospitals without an intensivist physician. Depending on the type of hospital, about 40% of patients were admitted to tertiary hospitals, and the mortality rate was higher in general hospitals (*n* = 5385, 12.2%) than in tertiary hospitals (*n* = 3,286, 11.4%). Mortality rates were high in regions other than capital and metropolitan areas, and the mortality rate was high in patients who had ventilators or central venous catheters inserted.

**Table 4:** Baseline characteristics of ICU inpatients

	Death		Survived		Total		*p*
	n/M	%/SD	n/M	%/SD	n/M	%/SD	
Quality assessment							
2nd	4236	(12.0)	31,078	(88.0)	35,314	(48.5)	.438
3rd	4435	(11.8)	33,130	(88.2)	37,565	(51.5)	
Intensivist physician staffing							
Yes	6167	(11.6)	47,037	(88.4)	53,204	(73.0)	< .001
No	2504	(12.7)	17,171	(87.3)	19,675	(27.0)	
Nurse-to-ICU bed ratio	0.76	± 0.52	0.74	± 0.51	0.74	± 0.51	< .001
Number of experienced nurses	45.09	± 42.33	49.64	± 46.94	49.10	± 46.44	< .001
Number of ICU specialized equipment	4.70	± 1.46	4.77	± 1.40	4.76	± 1.41	< .001
Number of ICU care protocols	8.89	± 0.90	8.92	± 0.70	8.92	± 0.73	< .001
Number of infection prevention bundles	3.07	± 0.96	3.13	± 0.91	3.12	± 0.91	< .001
Type of hospital							
Tertiary hospital	3286	(11.4)	25,459	(88.6)	28,745	(39.4)	.002
General hospital	5385	(12.2)	38,749	(87.8)	44,134	(60.6)	
Region							
Capital area	3539	(11.6)	26,894	(88.4)	30,433	(41.8)	.078
Metropolitan	2288	(11.9)	16,973	(88.1)	19,261	(26.4)	
Other	2844	(12.3)	20,341	(87.7)	23,185	(31.8)	
Number of beds							
< 300	1503	(12.9)	10,121	(87.1)	11,624	(15.9)	< .001
< 500	1310	(11.7)	9,933	(88.3)	11,243	(15.4)	
< 700	1489	(12.0)	10,923	(88.0)	12,412	(17.0)	
< 900	2144	(12.7)	14,738	(87.3)	16,882	(23.2)	
≥ 900	2225	(10.7)	18,493	(89.3)	20,718	(28.4)	
Sex							
Male	5111	(12.3)	36,455	(87.7)	41,566	(57.0)	< .001
Female	3560	(11.4)	27,753	(88.6)	31,313	(43.0)	
Age (years)	71.86	± 14.09	68.37	± 15.40	68.79	± 15.29	< .001
Ventilator application							
Yes	5850	(27.7)	15,249	(72.3)	21,099	(29.0)	< .001
No	2821	(5.4)	48,959	(94.6)	51,780	(71.0)	
Central venous catheter application							
Yes	4466	(25.5)	13,069	(74.5)	17,535	(24.1)	< .001
No	4205	(7.6)	51,139	(92.4)	55,344	(75.9)	
Health insurance							
NHI	7155	(12.0)	52,708	(88.0)	59,863	(82.1)	0.616
Medical aid	1504	(11.6)	11,413	(88.4)	12,917	(17.7)	
Veteran	12	(12.1)	87	(87.9)	99	(0.1)	
Disease							
Circulatory system (I00–I99)	2478	(10.3)	21,571	(89.7)	24,049	(33.0)	< .001
Respiratory system (J00–J99)	1858	(17.7)	8647	(82.3)	10,505	(14.4)	
Injury, poisoning, and certain other consequences of external causes (S00–T98)	707	(8.5)	7635	(91.5)	8342	(11.4)	
Digestive system (K00–K93)	612	(8.8)	6352	(91.2)	6964	(9.6)	
Genitourinary system (N00–N99)	444	(9.1)	4414	(90.9)	4858	(6.7)	
Malignant neoplasms (C00–C97)	1154	(18.6)	5040	(81.4)	6194	(8.5)	
Nervous system (G00–G99)	136	(6.4)	1988	(93.6)	2124	(2.9)	
Certain infections and parasitic diseases (A00–B99)	666	(20.0)	2661	(80.0)	3327	(4.6)	
Other	616	(9.5)	5900	(90.5)	6516	(8.9)	
Total	8671	(11.9)	64,208	(88.1)	72,879	(100)	

Table 5 shows the relationship between quality assessment and medical staff on mortality in ICU inpatients. Compared to the 2nd assessment, the 3rd assessment was associated with a decreased risk of in-hospital death (odds ratio [OR]: 0.950, 95% confidence interval [CI] 0.903–1.000, *p* = 0.048). Among medical institutions, hospitals with intensivist physicians were associated with reduced in-hospital mortality (OR: 0.842, 95% CI 0.769–0.924). A higher nurse-to-ICU bed ratio was associated with higher in-hospital deaths (OR: 1.056, 95% CI 1.001–1.114), and conversely, a more experienced nurse was associated with a lower risk of death (OR: 0.996, 95% CI 0.995–0.997). It was also associated with increased in-hospital mortality in patients who were on ventilators or with a central venous line.

**Table 5:** Relationship between quality assessments and medical staff on mortality in ICU inpatients

	OR	95% CI		*p*
Quality assessment				
2nd	1.000	–	–	
3rd	0.950	0.903	1.000	.048
Intensivist physician staffing				
Yes	0.842	0.769	0.924	< .001
No	1.000	–	–	
Nurse-to-ICU bed ratio	1.056	1.001	1.114	.045
Number of experienced nurses	0.996	0.995	0.997	< .001
Number of ICU specialized equipment	0.911	0.883	0.939	< .001
Number of ICU care protocols	0.983	0.953	1.013	.264
Number of infection prevention bundles	0.961	0.932	0.991	.012
Type of hospital				
Tertiary hospital	1.000	–	–	
General hospital	1.066	0.982	1.157	.128
Region				
Capital area	1.000	–	–	
Metropolitan	1.272	1.194	1.354	< .001
Other	1.072	1.007	1.141	.030
Number of beds				
< 300	1.172	1.023	1.341	.022
< 500	0.957	0.848	1.079	.471
< 700	0.993	0.891	1.106	.895
< 900	1.123	1.034	1.220	.006
≥ 900	1.000	–	–	
Sex				
Male	1.123	1.069	1.180	< .001
Female	1.000	–	–	
Age (years)	1.019	1.017	1.021	< .001
Ventilator application				
Yes	6.607	6.248	6.987	< .001
No	1.000	–	–	
Central venous catheter application				
Yes	2.620	2.491	2.756	< .001
No	1.000	–	–	
Health insurance				
NHI	1.000	–	–	
Medical aid	0.988	0.927	1.054	.721
Veteran	1.142	0.593	2.198	.692
Disease				
Circulatory system (I00–I99)	0.965	0.879	1.060	.463
Respiratory system (J00–J99)	1.116	1.008	1.235	.034
Injury, poisoning, and certain other consequences of external causes (S00–T98)	0.747	0.666	0.837	< .001
Digestive system (K00–K93)	1.002	0.892	1.125	.975
Genitourinary system (N00–N99)	0.879	0.772	1.000	.050
Malignant neoplasms (C00–C97)	1.914	1.715	2.137	< .001
Nervous system (G00–G99)	0.578	0.476	0.701	< .001
Certain infections and parasitic diseases (A00–B99)	1.849	1.633	2.093	< .001
Other	1.000	–	–	

**Fig. 1:**
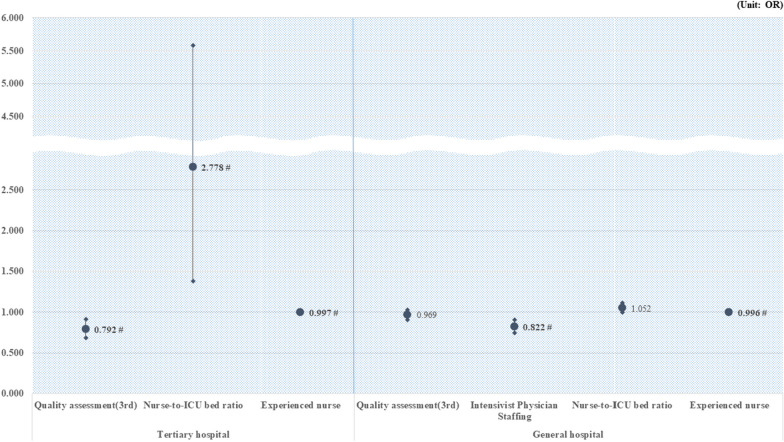
Subgroup results of the relationship between quality assessment and medical staff on mortality. Bold indicates significance

Figure 1 shows the impact of quality assessment and medical staff on mortality by hospital type. In the case of tertiary hospitals, in-hospital mortality decreased in the 3rd quality evaluation compared to the 2nd quality evaluation (OR: 0.792, 95% CI 0.686–0.913). An increase in nurse-to-ICU bed ratio was associated with an increase in in-hospital deaths but was significant in tertiary hospitals (OR: 2.778, 95% CI 1.382–5.584). In addition, as the number of experienced nurses increased, there was a decrease in in-hospital death in both general (OR 0.996, 95% CI 0.993–0.999) and tertiary hospitals (OR: 0.997, 95% CI 0.996–0.999).

## Discussion

Various efforts to improve the quality of care contribute to establishing standards for medical institutions. In South Korea, HIRA evaluates medical institutions based on various indicators. Specifically, appropriate management is important according to the severity of patients in the ICU, and better results can be expected through the application of standardized indicators. To evaluate these quality assessments’ effectiveness, this study evaluated changes in structural indicators in the ICU and their impact on patient outcomes.

First, quality assessment positively impacted structural indicators, including medical staff. Compared to the 2nd quality assessment, the 3rd quality assessment showed an increase in the number of intensivist physicians and a decrease in the nurse-to-bed ratio. These results suggest that the quality assessment made a positive contribution to the quantitative increase in staff in medical institutions. Similarly, previous studies have suggested that the pay-for-performance (P4P) program in ambulatory care is associated with improvements in the process of care and patient outcomes [19, 20]. However, some studies have not provided clear evidence of the cost-effectiveness of P4P, with some results suggesting that inequalities between socioeconomic groups have decreased, while other inequalities have generally been maintained [21]. Similarly, the gap between medical institutions observed in previous evaluations has remained unresolved. In the 3rd quality assessment, the average number of full-time intensive physicians in general hospitals was 0.4; however, it was 4 in tertiary hospitals, and the nurse-to-bed ratio was half that of tertiary hospitals. This is related to the imbalance in the distribution of medical resources and suggests that additional intervention is needed to reduce the gap between medical institutions by improving distribution.

Second, this study revealed that medical staff are an important factor in quality assessment indicators that could affect patient outcomes. Intensivist physicians, ICU specialized equipment, and infection prevention bundles were associated with a decrease in in-hospital mortality, and mortality increased as the nurse-to-bed ratio increased. Contrastingly, as the number of nurses with more than 3 years of experience increased, the in-hospital mortality of patients decreased significantly. These results suggest that having sufficient intensivists and nurses is important for better patient outcomes. Specifically, the presence of an intensivist is important considering the magnitude of its impact on in-hospital mortality, which is consistent with previous findings that the presence of a cardiac intensivist is associated with reduced mortality in adult cardiac care units [22]. However, some studies have not shown a significant association of the patient-to-intensivist ratio in reducing mortality [23, 24], suggesting that further research is needed to provide evidence on the impact of staffing levels on patient outcomes. In addition, the current results indicate that securing both sufficient and experienced nurses in the ICU is important. Nurses are the most direct healthcare workers, and they have an effect on patient outcomes; a shortage of nurses can increase nurses’ workload, which can negatively affect patient outcomes [25]. However, numerous new nurses are willing to quit or move to other jobs within 1–2 years, indicating the difficulty in securing nurses [26, 27]. The increase in experienced nurses through nurse retention is important for improving the care quality [28], and empowerment or changes in the medical environment are needed to lower nurses’ turnover rate [29, 30]. This study provides evidence that both experienced nurses and absolute nurse numbers are critical to achieving better outcomes for patients; thus, healthcare policymakers should find approaches to prevent new nurses from leaving. Finally, better patient outcomes can be expected through the provision of appropriate equipment and establishment of suitable ICU protocols.

The subgroup analysis results showed the effect of nurses on patient outcomes, according to the hospital type. In tertiary hospitals, an increase in the nurse-to-ICU bed ratio was associated with an increased risk of in-hospital death, and the number of experienced nurses was associated with decreased in-hospital death. These results were similar in general hospitals; however, only the increase in experienced nurses was associated with a significant decrease in in-hospital mortality. This could be related to the difference in the severity of patients in hospitals; this indicates that experienced nurses and the absolute number of nurses in ICUs are important in tertiary hospitals with critically ill patients.

In South Korea, various quality indicators are applied to manage the overall quality of medical institutions, the results are disclosed to the public, and incentives are provided to medical institutions within the compensation system according to the quality evaluation results. An incentive system linked to the quality evaluation of medical institutions will lead to profits for medical institutions and motivate them to meet the quality indicators. In addition, other factors, such as the reputation of a healthcare institution, can motivate healthcare institutions to participate in value-based payment systems and provide an opportunity to bring better value to patients [31]. In this study, quality assessment affected the improvement of overall structural indicators of medical institutions, which is related to the decreased risk of in-hospital deaths. This can be a positive aspect of introducing quality assessment; nevertheless, unresolved problems related to the distribution of resources in medical institutions still exist—an issue to be addressed in future research. Therefore, policymakers should strive for an equal distribution of medical resources, especially to increase experienced nurses and intensivist physicians. To this end, evaluating the overall quality level of medical institutions by continuously monitoring and applying the development of various indicators.

This study has several strengths. The data used in this study are representative; that is, all medical institutions subject to quality assessment of ICUs across the country were included. The current results inform policymakers, contribute to the improvement of ICU quality, and provide evidence that the application of quality indicators can improve patient outcomes. Furthermore, this study provides evidence for the impact on patients, wherein medical staff are structural indicators, and suggests the need for skilled medical staff for better patient outcomes. Finally, this study will be helpful for countries that want to develop and apply quality indicators within ICUs.

Despite these strengths, this study has some limitations. As this study included only quality assessment indicator data collected by the HIRA, assessing the clinical data or severity of patients admitted to the ICU was challenging owing to data constraints. Therefore, this study adjusted the insertion of a ventilator or central venous catheter, which could reflect patients’ severity. Moreover, as the ICU quality assessment only collects data for 3 months every 2 years, evaluating the effect on the outcome of long-term hospitalized patients, including those who were discharged during that period, was impossible. Therefore, further studies are needed on the long-term effects of quality assessment on patients hospitalized in an ICU for a long period or in medical institutions. Finally, other unmeasured factors, such as healthcare-associated infections, could influence patient mortality; thus, additional research is needed.

## Conclusions

This study found a positive impact of ICU quality assessments on medical institutions. The introduction of ICU quality assessments improved the overall structural indicators of medical institutions, and improvements in these indicators led to positive patient outcomes. Specifically, medical personnel affect in-hospital mortality, and securing skilled medical staff is important for medical institutions to ensure better patient outcomes. However, gaps between medical institutions still exist even after quality assessments, and additional policy interventions are needed to solve these problems. This study provides evidence for improving the quality assessment program, and long-term monitoring and evaluation are needed to improve the quality of ICUs.

## Data Availability

The data that support the current findings are available from the HIRA Service. However, restrictions apply to the availability of these data, which were used under license for this study; thus, they are not publicly available. Data are, however, available from the authors upon reasonable request and with permission of the HIRA Service: (https://opendata.hira.or.kr/home.do).

## Abbreviations

HIRA: Health Insurance Review and Assessment Service
ICU: Intensive care unit
NHI: National Health Insurance
P4P: Pay-for-performance
